# Dietary intake, antioxidants, minerals and vitamins in relation to childhood asthma: a Mendelian randomization study

**DOI:** 10.3389/fnut.2024.1401881

**Published:** 2024-05-23

**Authors:** Liang Luo, Guanglei Chen, Yan Zhou, YaJun Xiang, Jing Peng

**Affiliations:** ^1^School of TCM Health Care, Leshan Vocational of Technical College, Leshan, Sicuan Province, China; ^2^School of Basic Medicine, Guizhou University of Traditional Chinese Medicine, Guiyang, Guizhou Province, China

**Keywords:** childhood asthma (CA), daily dietary intake, vitamins, minerals, antioxidants, Mendelian randomization (MR)

## Abstract

**Background:**

Currently, there is limited and inconsistent evidence regarding the risk association between daily dietary intake, antioxidants, minerals, and vitamins with Childhood Asthma (CA). Therefore, this study employs Mendelian Randomization (MR) methodology to systematically investigate the causal relationships between daily dietary intake, serum antioxidants, serum minerals, and the circulating levels of serum vitamins with *CA.*

**Methods:**

This study selected factors related to daily dietary intake, including carbohydrates, proteins, fats, and sugars, as well as serum antioxidant levels (lycopene, uric acid, and β-carotene), minerals (calcium, copper, selenium, zinc, iron, phosphorus, and magnesium), and vitamins (vitamin A, vitamin B6, folate, vitamin B12, vitamin C, vitamin D, and vitamin E), using them as Instrumental Variables (IVs). Genetic data related to CA were obtained from the FinnGen and GWAS Catalog databases, with the primary analytical methods being Inverse Variance Weighting (IVW) and sensitivity analysis.

**Results:**

Following MR analysis, it is observed that sugar intake (OR: 0.71, 95% CI: 0.55–0.91, P: 0.01) is inversely correlated with the risk of CA, while the intake of serum circulating magnesium levels (OR: 1.63, 95% CI: 1.06–2.53, P: 0.03), fats (OR: 1.44, 95% CI: 1.06–1.95, P: 0.02), and serum vitamin D levels (OR: 1.14, 95% CI: 1.04–1.25, P: 0.02) are positively associated with an increased risk of *CA.*

**Conclusion:**

This study identified a causal relationship between the daily dietary intake of sugars and fats, as well as the magnesium and vitamin D levels in serum, and the occurrence of *CA.* However, further in-depth research is warranted to elucidate the specific mechanisms underlying these associations.

## 1 Introduction

Asthma is a prevalent chronic respiratory disease, particularly common in children. Its characteristics encompass airway inflammation, recurrent wheezing, and heightened bronchial reactivity, resulting in airway constriction and obstructed airflow. Clinical symptoms include wheezing, coughing, and shortness of breath. The global incidence of asthma is on the rise, with an estimated affected population exceeding 300 million (1). In the United States, asthma prevalence is relatively high, impacting over 25 million individuals, with approximately 9 million children affected by childhood asthma (CA) (2). Major risk factors for CA include exposure to cigarette particles (3) and air pollution (4). However, dietary also play a role in the occurrence and development of CA, a diversified diet can reduce the risk of CA by improving the intestinal flora (5). There exists a complex relationship between allergic diseases and nutritional status (6). Therefore, elucidating their causal relationships with CA holds significant implications for the prevention and treatment of this condition.

Mendelian randomization (MR) is a research method employed to analyze the relationship between exposure risk factors and disease outcomes. It utilizes genetic variants as instrumental variables (IVs) to substitute for correlated risk factors (7). By doing so, it assesses causal relationships between exposure factors and outcomes, as the alleles are randomly assigned during conception, making genetic variations less susceptible to measurement bias or confounding factors such as reverse causation.

This study aimed to employ MR analysis to ascertain the causal relationships between nutrient intake levels (fat, protein, sugar, and carbohydrates) and the serum circulating concentrations of 17 micronutrients (antioxidants, minerals, and vitamins) with *CA.* A meta-analysis was conducted for predictive purposes, with the ultimate goal of contributing to the prevention and treatment of *CA.*

## 2 Materials and methods

### 2.1 Study design

**Figure 1:**
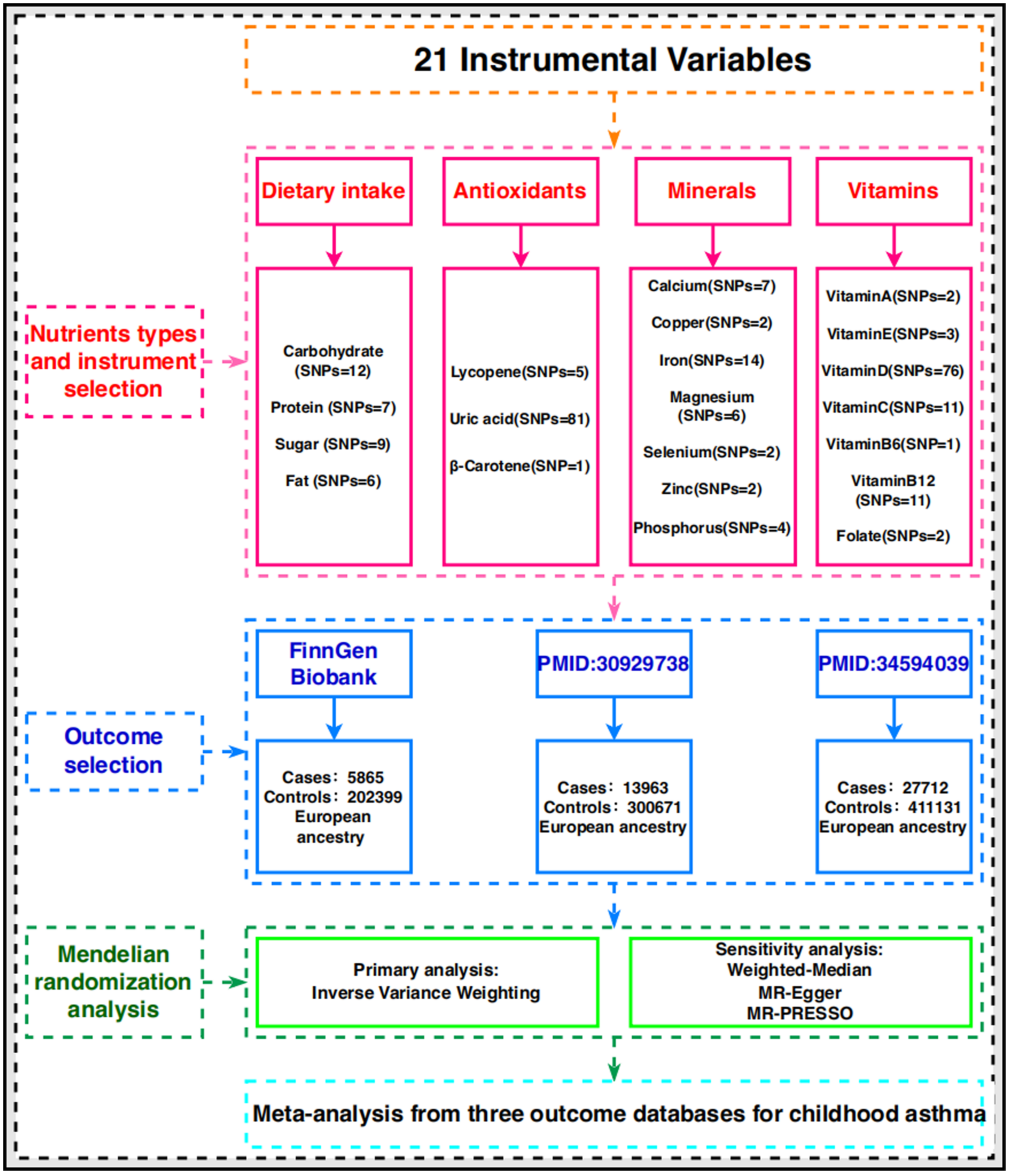
A flowchart of study design.

The design of our MR study is illustrated in Figure 1. We utilized publicly available genome-wide association study (GWAS) data from the FinnGen and GWAS Catalog databases for various exposure-related MR analyses. Subsequently, we conducted a meta-analysis of MR results from different databases, providing a comprehensive assessment of the associations between each exposure and risk. We applied the Benjamin-Hochberg correction method to conduct multiple independent tests for CA, correcting for the false discovery rate (FDR) in multiple testing. Only associations with Benjamin-Hochberg corrected *p*-values less than 0.05 were considered significant. Pooled statistics from publicly available studies were used in this study, so we did not need to obtain any additional ethical approvals.

### 2.2 Data sources

In this MR study, CA serves as the primary outcome, and Table 1 provides a comprehensive overview of GWAS data for CA sourced from three distinct databases. Table 2 succinctly delineates the daily dietary intake, antioxidants, minerals, and vitamins employed as instrumental variables (IVs). It is noteworthy that all GWAS data used in this study originated from populations of European descent.

**Table 1:** Genome-wide association study data profiles for the outcome variables used in this study.

Outcome	Trait	Sample description	Consortium	PMID	Website	Abbreviate
CA	age < 16	5,865 European ancestry cases, 202,399 European ancestry controls	Finngen Biobank	-	https://r9.finngen.fi/pheno/ASTHMA_CHILD_EXMORE	FCA
CA	childhood onset asthma	13,963 European ancestry cases, 300,671 European ancestry controls	-	30,929,738	https://www.ebi.ac.uk/gwas/studies/GCST007800	1COA
CA	childhood onset asthma	27,712 European ancestry cases, 411,131 European ancestry controls, 547 East Asian ancestry cases, 161,803 East Asian ancestry controls	-	34,594,039	https://www.ebi.ac.uk/gwas/studies/GCST90018895	2COA

**Table 2:** Genome-wide association study data profiles for IVs used in this study.

Exposure	SNP	Sample Size	Ethnicity	R^2*^	F**	PMID
Carbohydrate (8)	12	268,922	European	0.18%	39.5	32,393,786
Fat (8)	6	268,922	European	0.13%	58.8	32,393,786
Protein (8)	7	268,922	European	0.14%	53.7	32,393,786
Sugar (8)	9	235,391	European	0.19%	48.7	32,393,786
β-carotene (9)	1	3,881	European	2.48%	98.6	19,185,284
Lycopene (10)	5	441	European	31.01%	39.1	26,861,389
Uric acid (11)	81	288,649	European	2.33%	85.1	31,578,528
Calcium (12)	7	60,958	European	0.84%	73.9	24,068,962
Copper (13)	2	5,594	European	1.94%	55.4	34,523,676
Selenium (14)	2	9,639	European	2.12%	104.3	25,343,990
Iron (15)	14	163,511	European	2.63%	314.9	33,536,631
Magnesium (16)	6	23,829	European	1.45%	58.5	20,700,443
Phosphorus (17)	4	21,807	European	0.75%	41.1	20,558,539
Zinc (18)	2	2,603	European	4.59%	62.6	23,720,494
Vitamin A (19)	2	8,902	European	0.63%	28.4	21,878,437
Folate (20)	2	37,337	European	0.76%	142.1	23,754,956
Vitamin B12 (20)	11	37,283	European	5.13%	183.3	23,754,956
Vitamin B6 (21)	1	4,763	European	1.02%	49.0	19,744,961
Vitamin C (22)	11	52,018	European	1.79%	86.0	33,203,707
Vitamin D (23)	76	401,529	European	3.68%	201.8	33,431,812
Vitamin E (24)	3	8,781	European	0.39%	11.4	21,729,881

*R^2^ were calculated as R^2^ = 2 * Beta^2^ * EAF * (1-EAF) / (2 * Beta^2^ * EAF * (1-EAF) + SE^2^* 2 * Sample Size * EAF * (1-EAF); **F-statistics were calculated as F = R^2^ * (Sample Size - 1 - k) / ((1-R^2^) * k). Details of the specific Beta, EAF, SE, sample size and k values for each SNP are shown in Supplementary material 1).

### 2.3 Data integration

Given that genetic variations are formed randomly at the time of maternal conception and are independent of environmental factors, MR analysis is less susceptible to reverse causation and confounding compared to traditional observational methods. In this study, MR analysis was employed to ascertain the relationship between Single Nucleotide Polymorphisms (SNPs) associated with daily dietary intake, antioxidants, minerals, and vitamins and the risk of *CA.* When selecting SNPs as instrumental variables (IVs), three criteria must be satisfied: (1) each IV is significantly correlated with the corresponding levels of daily dietary intake, antioxidants, minerals, and vitamins. (2) Each IV influences CA solely through the pathways of daily dietary intake, antioxidants, minerals, and vitamins. (3) Each IV is not influenced by confounding factors, reducing bias introduced by Linkage Disequilibrium (LD) among SNPs. The inclusion criteria for IVs are adapted from previous studies (25, 26): adopting a genome-wide significance threshold (*p* < 5 × 10^−8^). Furthermore, the physical distance between any two genes must exceed 10,000 kb, and the R^2^ threshold for LD between genes is set at <0.001. Finally, SNPs with an F-statistic greater than 10 are defined as strong instrumental variables, while those with an F-statistic less than 10 are considered weak instrumental variables and are excluded.

### 2.4 Statistical analysis

The Mendelian Randomization analysis was conducted using R (version 4.3.1) and the R package “Two Sample MR” (version 0.5.7) (27). In this MR analysis, the primary method employed was the Inverse Variance Weighting (IVW) (28) approach, assessing the association between each dietary intake or nutritional element and *CA.* Individual instrumental variables (IVs), such as selenium and vitamin B6, utilized the Wald Ratio (WR) to estimate their effects. As all Single Nucleotide Polymorphisms (SNPs) were considered valid IVs, the IVW method provided robust estimates of causal effects. Meta-analysis was performed using the R package “meta” (version 6.5.0), predominantly employing a fixed-effects model to synthesize risk estimates for each exposure, facilitating an overall risk assessment and prediction.

In cases where a minimum of three valid Instrumental Variables (IVs) were available, we conducted sensitivity analyses employing three alternative MR methods to explore potential biases introduced by ineffective IVs. The sensitivity analysis methodologies encompassed MR-Egger regression (MRE) (29), Weighted-median (WM) (30), and Mendelian Randomization Pleiotropy RESidual Sum and Outlier (MR-PRESSO) (31). Sensitivity to horizontal pleiotropy was addressed primarily through the utilization of MR-PRESSO (31) upon the detection of conspicuous outliers, given its capability not only to identify SNP outliers but also to correct for them. Furthermore, when more than 50% of the total weight was attributed to effective IVs, WM (30) was employed, providing precise estimates of causal relationships. To account for transverse pleiotropy, if the directional effect estimates from MR-Egger (29) aligned with those of IVW, the former was considered valid. Evaluation metrics employed included Odds Ratios (OR) and their corresponding 95% Confidence Intervals (CI). Statistical significance was asserted when *p* < 0.05.

## 3 Results

**Figure 2:**
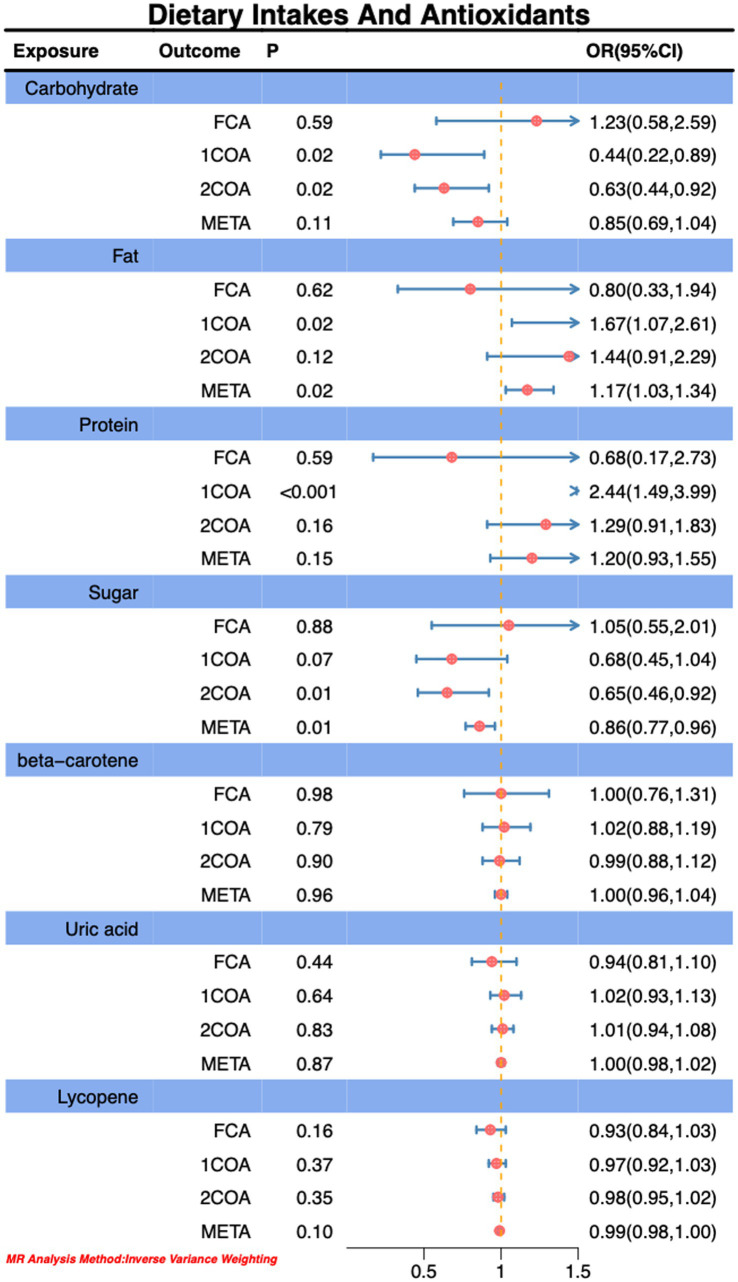
Association of dietary intakes and antioxidants with risk of *CA.*

**Figure 3:**
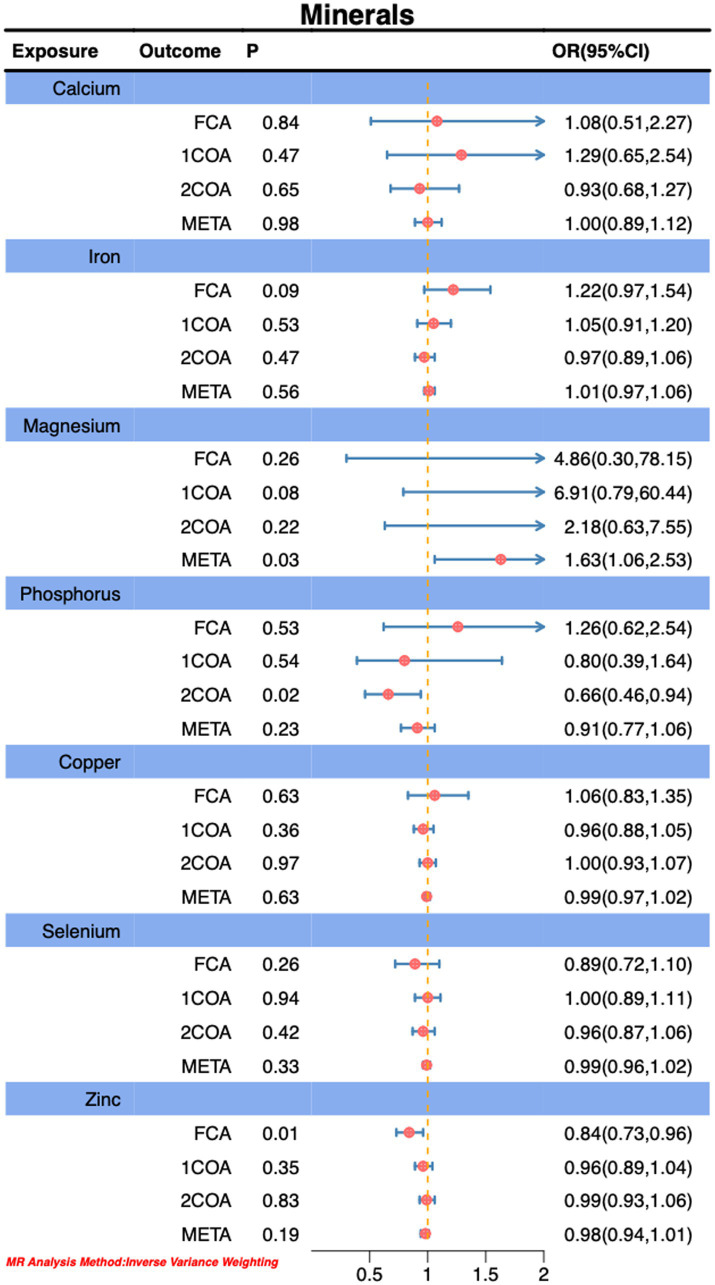
Association of Minerals with risk of *CA.*

**Figure 4:**
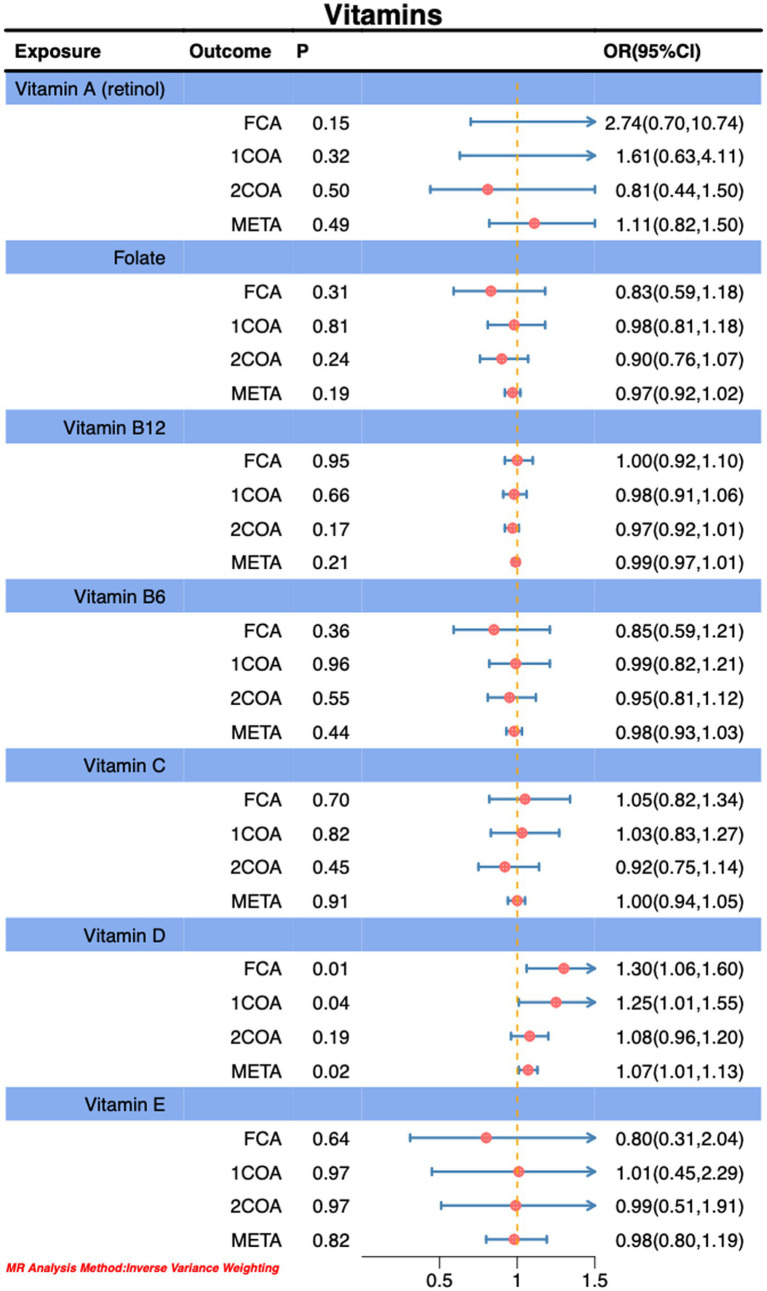
Association of vitamins with risk of *CA.*

In this Mendelian randomization analysis, we observed that genetically predicted higher sugar intake (OR: 0.71, 95% CI: 0.55–0.91, P: 0.01) is associated with the risk reduction of CA, as depicted in Figure 2. However, we also noted that genetically predicted higher daily fat intake (OR: 1.44, 95% CI: 1.06–1.95, P: 0.02) is associated with an increased risk of CA, as illustrated in Figure 2. Notably, our findings revealed a positive correlation between genetically predicted serum vitamin D levels (OR: 1.14, 95% CI: 1.04–1.25, P: 0.02) and serum circulating magnesium levels (OR: 1.63, 95% CI: 1.06–2.53, P: 0.03) with the risk of *CA.* This observation is validated across three additional databases, as shown in Figures 3, 4. The overall trend in sensitivity analysis aligns with these findings.

During MR-PRESSO analysis of carbohydrates, proteins, and vitamin D, some outliers were detected. However, upon their exclusion, the observed associations remained unchanged, as detailed in the Supplementary material. Consequently, our study results suggest an inverse correlation between higher sugar intake and the risk of CA, while higher fat intake, circulating magnesium levels, and serum vitamin D levels are positively associated with an increased risk of *CA.* Importantly, this association persists even after accounting for potential analytical outliers.

## 4 Discussion

Through MR analysis, we observed a significant negative correlation between genetically predicted sugar intake and the risk of CA, while fat intake, circulating magnesium levels, and serum vitamin D levels exhibited positive correlations with CA risk. Meta-analysis further confirmed the statistical significance of these associations, aligning with the results from some databases (Figures 2–4).

Early observational studies extensively explored the relationship between daily dietary intake and susceptibility to *CA.* However, this MR analysis reveals a contradiction in the impact of sugar intake on CA compared to prior research (32). Nevertheless, other association outcomes align with early investigations (33, 34). The International Study of Asthma and Allergies in Childhood (ISAAC) highlights the protective effect of the Mediterranean Diet (MD) on CA during early childhood (35, 36). This diet, primarily comprising carbohydrates such as grains, nuts, and legumes (37), may mitigate airway inflammation and related symptoms by modulating asthma-associated factors such as IL-4, IL-33, and IL-17 (38, 39). The MD also contributes to improving inflammatory markers such as high-sensitivity C-reactive protein and adiponectin (36), thus alleviating bronchial hyperresponsiveness. Concerning the relationship between sugar intake and CA, studies have established a positive correlation between sugar intake and the global incidence of CA (40–43). Recent meta-analyses further support these findings (44). Fructose, a primary component of added sugars in fruit juices and beverages, is associated with metabolic disturbances and asthma-like symptoms in non-obese mice (45) and may contribute to CA development by inducing inflammation (46). Food additives in sugary drinks, such as sodium benzoate or sulfites, may trigger urticaria and contact dermatitis, thereby inciting asthma (47). Although the relationship between fat and protein intake and CA remains unclear, a high-fat, high-protein diet may increase the risk of CA in children by promoting obesity (48). Relevant meta-analyses emphasize the pivotal role of overweight or obesity in CA risk (49) and elucidate that a high-fat diet may lead to an increase in circulating fatty acids (50), subsequently triggering inflammation and releasing TNF-α and IL-6, thereby promoting CA development (51). Excessive intake of fat and glucose may induce endoplasmic reticulum stress, initiate the unfolded protein response, and further activate inflammatory pathways (52). In summary, disparities between observational studies and MR analysis may arise from various reasons. Nevertheless, our research results indicate that increasing the intake of complex carbohydrates in the MD pattern may be one of the ways that the MD pattern can alleviate *CA.*

This study did not substantiate a significant association between serum antioxidants and *CA.* Nevertheless, earlier investigations have indeed revealed a distinct correlation between serum antioxidant levels and dietary antioxidant intake, potentially stemming from the intricate interplay between serum antioxidants and dietary antioxidant consumption (53, 54). The primary focus of this MR methodology is on serum antioxidants as the exposure, while overlooking dietary antioxidant intake as an exposure, which could be one of the contributing factors to the observed bias. Antioxidants effectively neutralize free radicals by providing electrons to alleviate oxidative damage (55). In the context of oxidative stress (OS), reactive oxygen species (ROS) are generated by immune cells, accompanied by impaired antioxidant reactions that exacerbate oxidative stress, leading to tissue damage and promoting airway inflammation and hyperreactivity (56). Key dietary antioxidants include vitamin E, vitamin C, carotenoids, ubiquinone, flavonoids, and selenium. Certain carotenoids such as α-carotene, β-cryptoxanthin, lutein/zeaxanthin, and lycopene exhibit a close relationship with lung function. For instance, reduced intake of lutein/zeaxanthin is associated with a decline in lung function (57), and the mechanistic action may involve β-carotene neutralizing highly reactive superoxide anions and directly interacting with peroxide radicals, thereby inhibiting the cascade of oxidative damage (58). In summary, although the direct association between antioxidants and CA remains inconclusive in this MR analysis, previous research underscores the regulatory role of supplemented antioxidants in mitigating oxidative damage. This underscores the significance of antioxidants in the prevention and management of *CA.* Consequently, further investigations are warranted to elucidate the intricate relationships among serum antioxidants, dietary antioxidant intake, and CA risk.

In this MR study, we observed significant associations of phosphorus and zinc in one database, while magnesium, although showing significance in the meta-analysis results of this MR analysis, did not exhibit a consistent trend in three other databases. Previous investigations have compared copper, zinc, magnesium, iron, and calcium levels in 40 Bronchial asthma (BA) patients and 43 healthy individuals, revealing significantly elevated copper and calcium levels in the asthma group (*p* < 0.001), while zinc levels in the healthy group exhibited a marked decrease (*p* < 0.01) (59). Another Japanese study found a significant increase in serum zinc levels in female asthma patients, with a positive correlation between serum zinc levels and regulatory activity (60). Allergic asthma patients showed a slight increase in serum copper concentration compared to healthy individuals (61). Assessment of lung function in BA patients revealed increased expression of superoxide dismutase and its associated genes, indicating enhanced oxidative stress (62). Utilizing atomic absorption techniques to study trace elements in the serum of asthma patients, elevated copper and iron concentrations and reduced magnesium and manganese concentrations were observed, suggesting a potential association of these elements with the pathogenesis of asthma (63). Further research in asthma-chronic obstructive pulmonary disease (ACO) demonstrated a potential impact of serum magnesium on lung function (64). Spectrophotometric determination of extracellular and intracellular magnesium concentrations revealed a strong positive correlation between intracellular magnesium levels and bronchial hyperresponsiveness. The mechanism might be linked to magnesium’s crucial role in calcium transport mechanisms and intracellular phosphorylation reactions, influencing the contraction and relaxation of bronchial smooth muscles, thereby leading to bronchial hyperresponsiveness and the development of asthma (65). However, some studies indicated that no significant association exists between serum magnesium and asthma symptom control in mildly asthmatic children aged 6–12 years (66). Significant differences exist in serum mineral levels and mineral intake concerning asthma. For instance, oral magnesium supplementation can reduce asthma symptoms (67), and magnesium and potassium intake correlates with childhood lung function (68). Overall, although our MR analysis revealed a positive correlation between serum magnesium and the risk of CA, we were unable to establish specific causal relationships between serum copper, phosphorus, and CA risk. However, the results of observational studies are generally consistent with those of the MR analysis, and any discrepancies may be attributed to differences in sample size, disease subtypes, or databases.

In our MR investigation, a significant association was uncovered between genetically predicted serum vitamin D levels and the risk of *CA.* Despite extensive exploration of the interaction between vitamin D and CA in previous studies, the relationship remains intricate. The human body acquires vitamin D primarily through two pathways: synthesis in the skin under sunlight, converting 7-dehydrocholesterol into vitamin D, and dietary intake. Vitamin D receptors are widely distributed in various tissues, playing a crucial role in numerous physiological processes (69, 70). Research also suggests that the prenatal impact of vitamin D on early-onset asthma appears to be linked to genetic variations in 17q21, the vitamin D receptor, and the vitamin D binding protein (71, 72). Vitamin D supplementation may potentially reduce the risk of neonatal asthma and have a positive effect on mitigating acute asthma exacerbations in adults with low 25-hydroxyvitamin D levels (73). The underlying mechanism may involve immune pathway modulation and interaction with various cells to alleviate asthma inflammation (74). Despite recent research indicating a correlation between lower levels of 25-OHD and CA, with evidence suggesting that vitamin D supplementation can reduce the progression of CA (75), the role of vitamin D in CA risk remains inconclusive. Prospective studies also cast doubt on the efficacy of vitamin D supplementation in enhancing the control of CA (69). Randomized, double-blind, placebo-controlled trials have also suggested that vitamin D3 supplementation does not significantly shorten the duration of severe asthma attacks (76). Comprehensive reviews of observational studies examining the preventive effects of vitamin D in infancy on asthma and wheezing have yielded inconsistent results. There is no direct causal relationship found between vitamin D intake in healthy infants and the occurrence of asthma (8, 9, 77, 78). In summary, the academic community exhibits diverse perspectives on the role of vitamin D in CA, necessitating further in-depth research to arrive at definitive conclusions.

The strength of this MR study lies in the integration of GWAS data from three independent databases, subjected to a meticulous meta-analysis, elucidating the genetic factors associated with *CA.* In contrast to previous investigations, this study not only scrutinizes micronutrients in serum but extends its focus to dietary intake, comprehensively assessing their intricate relationships with *CA.* However, certain limitations warrant consideration: the analysis is based on samples of European and East Asian ancestry, posing a potential risk of bias; despite employing the most comprehensive GWAS dataset to identify instrumental variables (IVs), these IVs have inherent limitations in their explanatory scope, and the scale of cohorts may inadequately influence the precision of IV selection. Future endeavors necessitate additional GWAS studies on trace elements to refine IV selection. Meanwhile, this study did not investigate the relationship between dietary intake levels and CA, which could be a potential direction for further research.

## 5 Conclusion

The primary findings of this study suggest that an increase in sugar intake, coupled with a decrease in dietary fat content, may be associated with a reduced risk of *CA.* Furthermore, MR analysis revealed a significant correlation between elevated levels of serum circulating magnesium and serum vitamin D and an increased risk of *CA.* However, these results are derived from MR analysis and may require further investigation to validate these associations and gain deeper insights into the potential mechanisms linking dietary intake and trace elements with the risk of *CA.* This MR study provides robust support for the association between dietary intake, micronutrients, and CA through the integration of multiple databases. Nevertheless, inherent limitations, such as population selection and the explanatory power of IVs, and the lack of research on intake levels. These limitations underscore the need for further research.

## Data availability statement

The datasets presented in this study can be found in online repositories. The names of the repository/repositories and accession number(s) can be found in the article/Supplementary material.

## Ethics statement

Ethical approval was not required for the studies involving humans because Pooled statistics from publicly available studies were used in this study, so we did not need to obtain any additional ethical approvals. The studies were conducted in accordance with the local legislation and institutional requirements. Written informed consent for participation was not required from the participants or the participants’ legal guardians/next of kin in accordance with the national legislation and institutional requirements because Pooled statistics from publicly available studies were used in this study, so we did not need to obtain any additional ethical approvals.

## Author contributions

LL: Conceptualization, Formal analysis, Methodology, Project administration, Writing – original draft, Writing – review & editing, Data curation, Funding acquisition, Investigation, Resources, Software. GC: Formal analysis, Investigation, Methodology, Software, Visualization, Data curation, Resources, Supervision, Validation, Writing – review & editing. YZ: Investigation, Project administration, Supervision, Validation, Writing – review & editing. YX: Project administration, Resources, Supervision, Validation, Visualization, Writing – review & editing. JP: Formal analysis, Funding acquisition, Investigation, Resources, Supervision, Visualization, Writing – review & editing.
