# Supplementary material for: Dietary intake, antioxidants, minerals and vitamins in relation to childhood asthma: a Mendelian randomization study
Source: Front Nutr. 2024 May 23;11:1401881. doi: 10.3389/fnut.2024.1401881 (PMC11153797; doi:10.3389/fnut.2024.1401881)

Exposure：Carbohydrate；Outcome：FinnGen(childhood asthma)-30929738(childhood asthma)-34594039(childhood asthma)


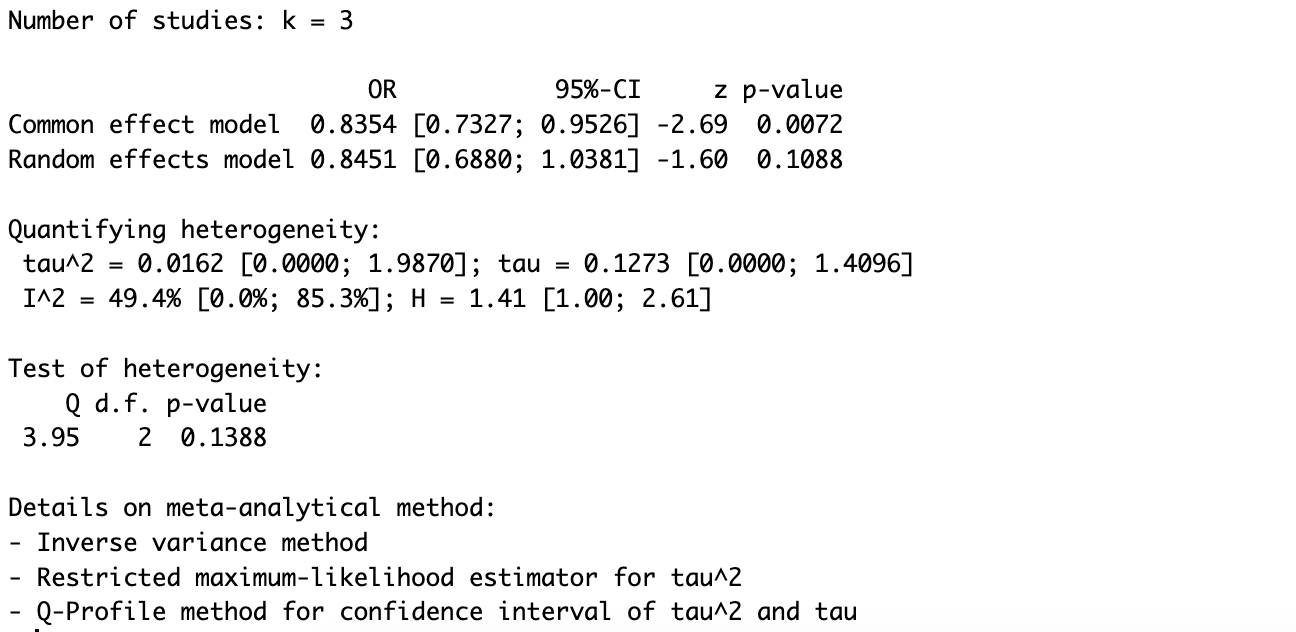


Exposure：Fat；Outcome：FinnGen(childhood asthma)-30929738(childhood asthma)-34594039(childhood asthma)


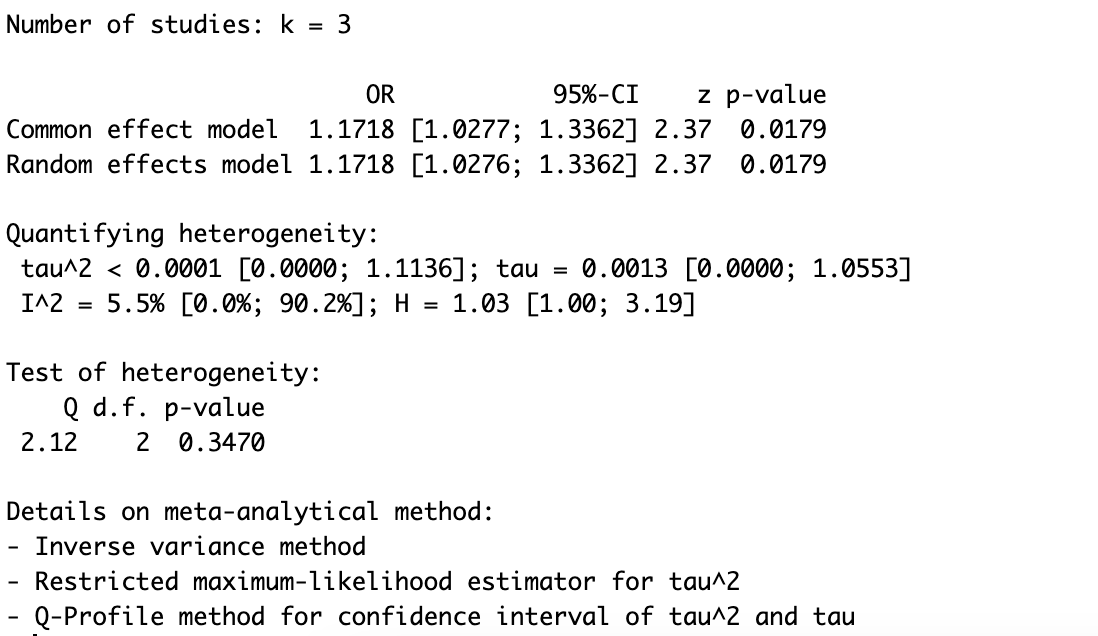


Exposure：Protein；Outcome：FinnGen(childhood asthma)-30929738(childhood asthma)-34594039(childhood asthma)


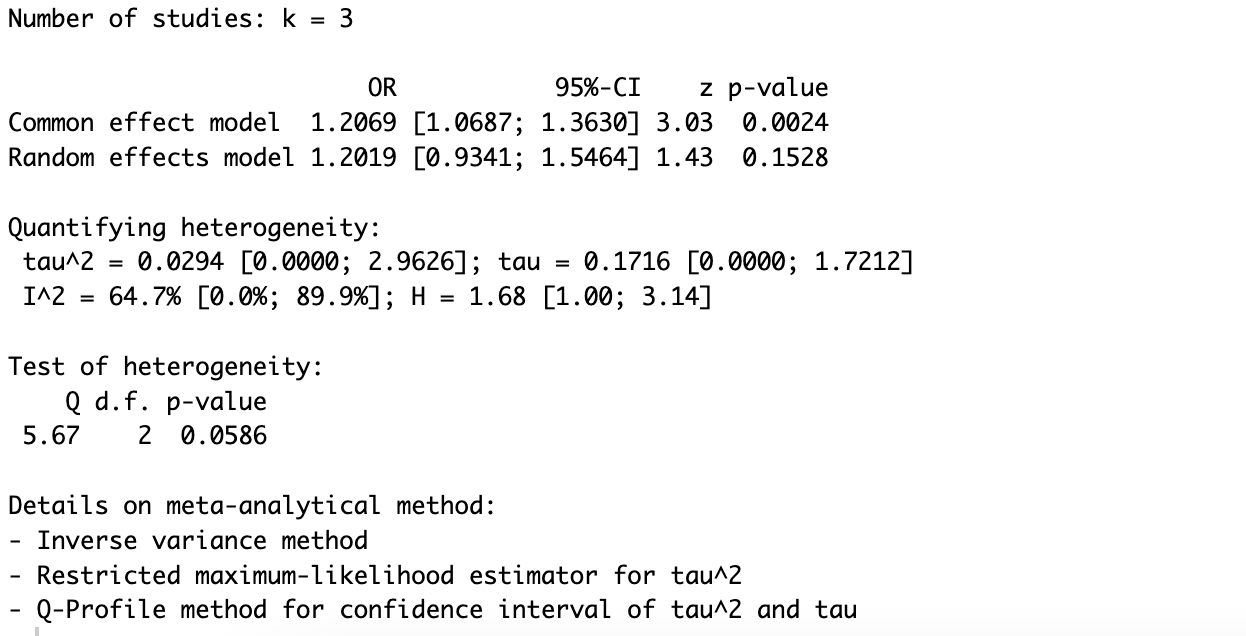


Exposure：Sugar；Outcome：FinnGen(childhood asthma)-30929738(childhood asthma)-34594039(childhood asthma)
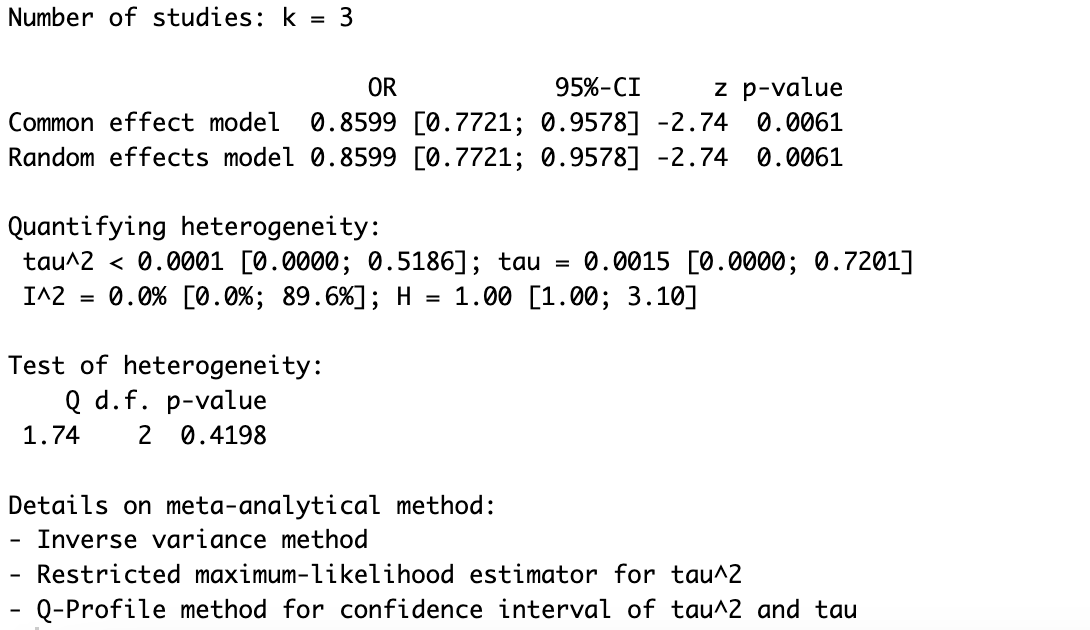


Exposure：β-carotene；Outcome：FinnGen(childhood asthma)-30929738(childhood asthma)-34594039(childhood asthma)


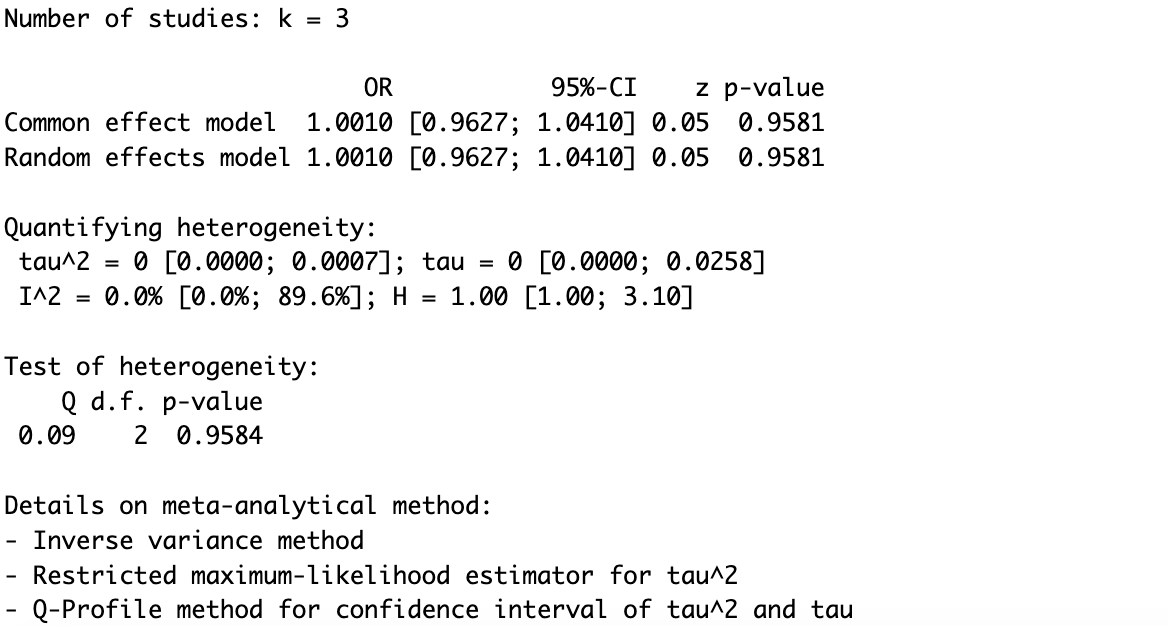


Exposure：Uric acid；Outcome：FinnGen(childhood asthma)-30929738(childhood asthma)-34594039(childhood asthma)


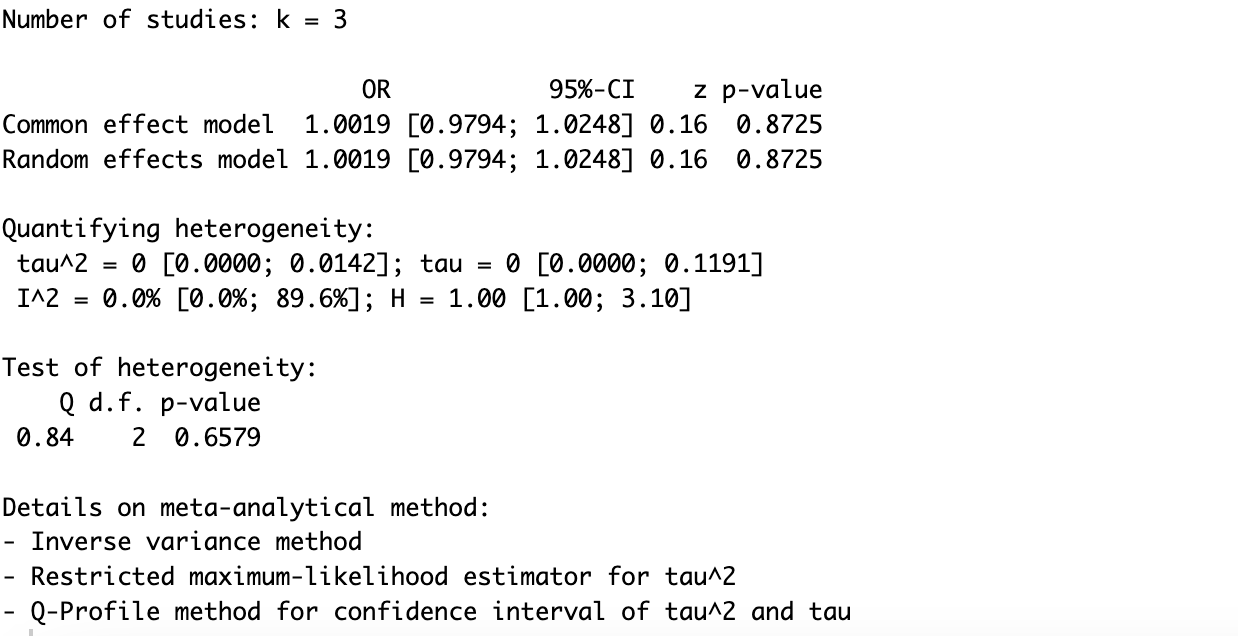


Exposure：Lycopene；Outcome：FinnGen(childhood asthma)-30929738(childhood asthma)-34594039(childhood asthma)


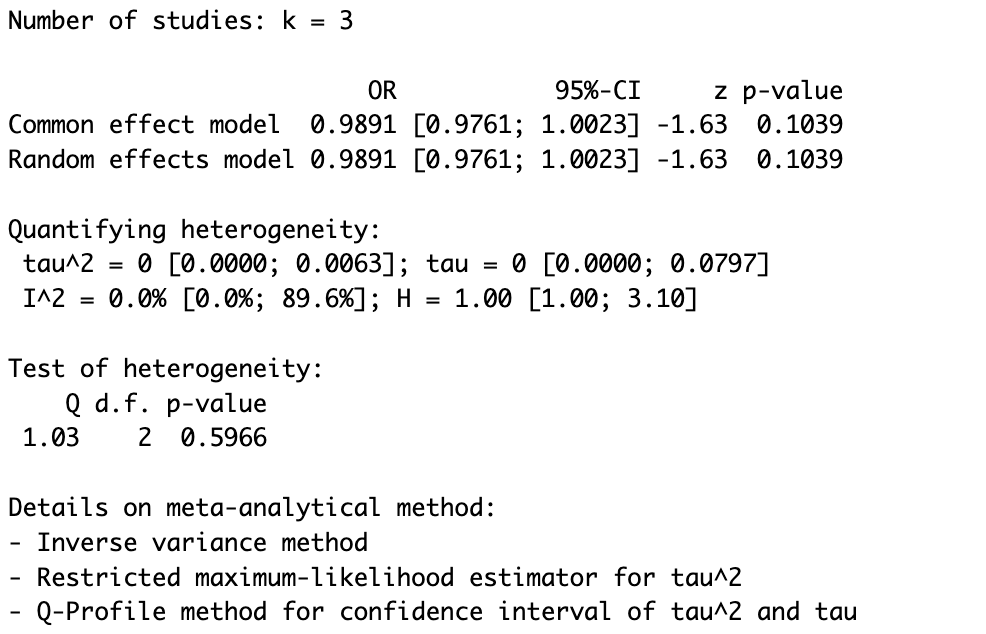


Exposure：Calcium；Outcome：FinnGen(childhood asthma)-30929738(childhood asthma)-34594039(childhood asthma)


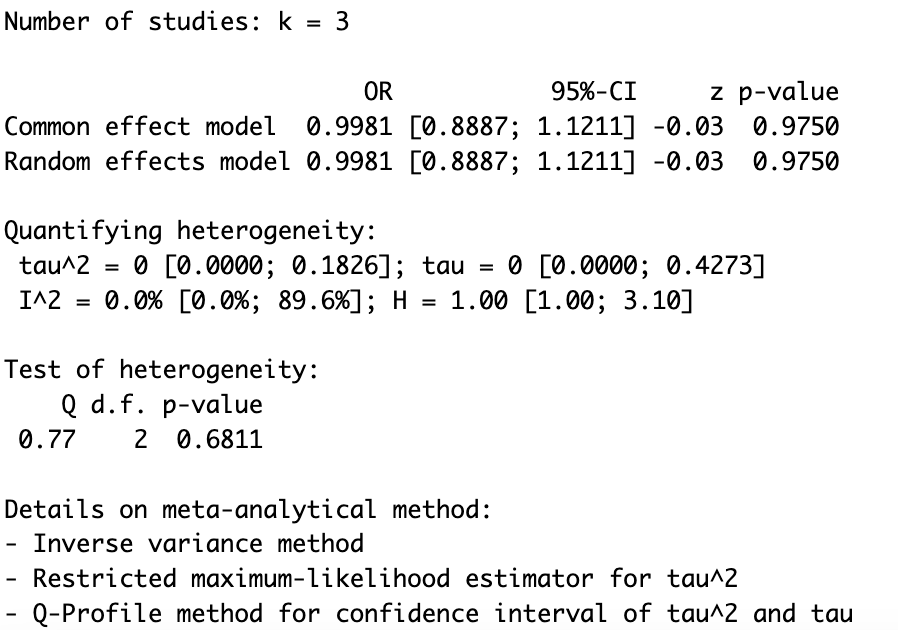


Exposure：Iron；Outcome：FinnGen(childhood asthma)-30929738(childhood asthma)-34594039(childhood asthma)


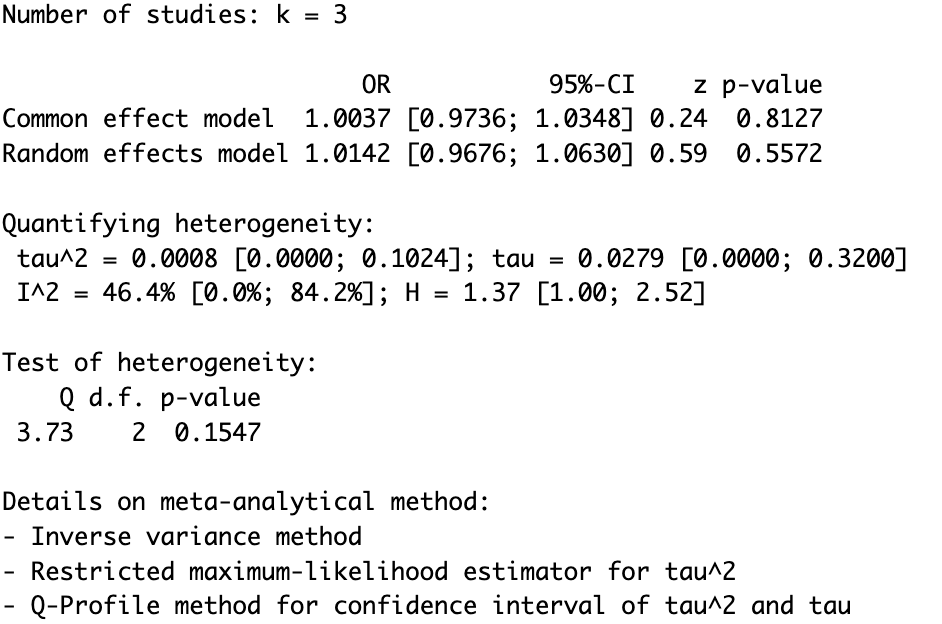


Exposure：Magnesium；Outcome：FinnGen(childhood asthma)-30929738(childhood asthma)-34594039(childhood asthma)


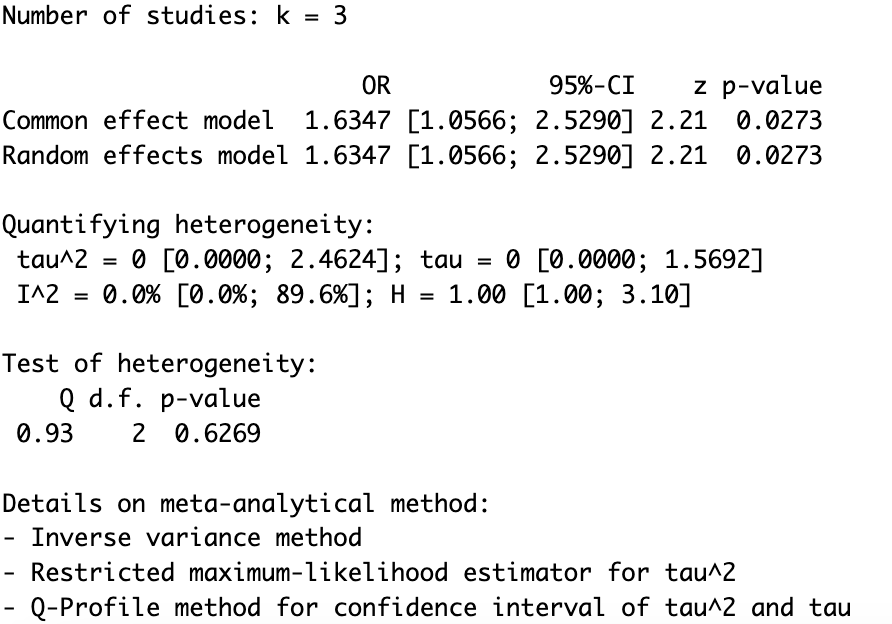


Exposure：Phosphorus；Outcome：FinnGen(childhood asthma)-30929738(childhood asthma)-34594039(childhood asthma)


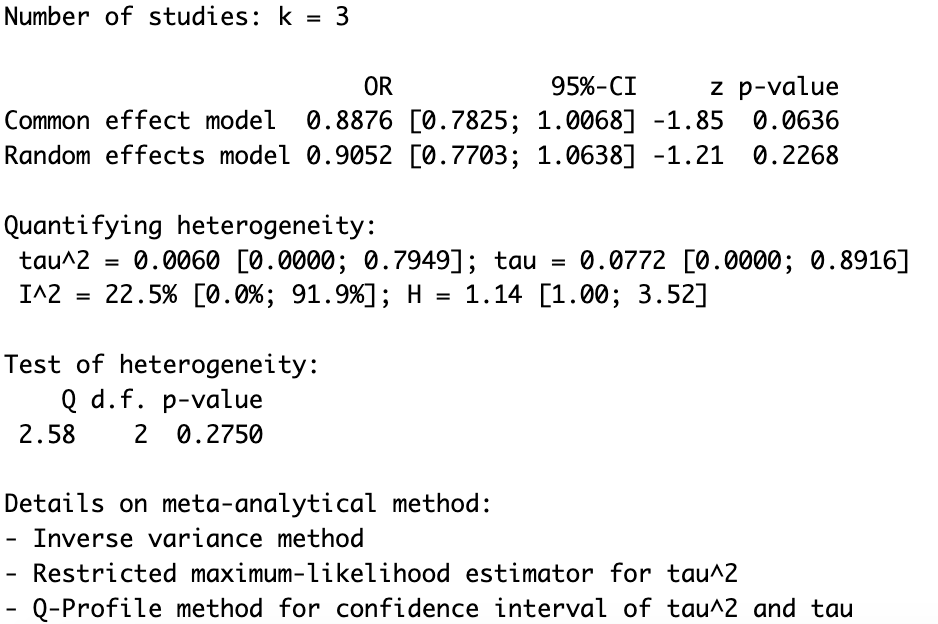


Exposure：Copper；Outcome：FinnGen(childhood asthma)-30929738(childhood asthma)-34594039(childhood asthma)


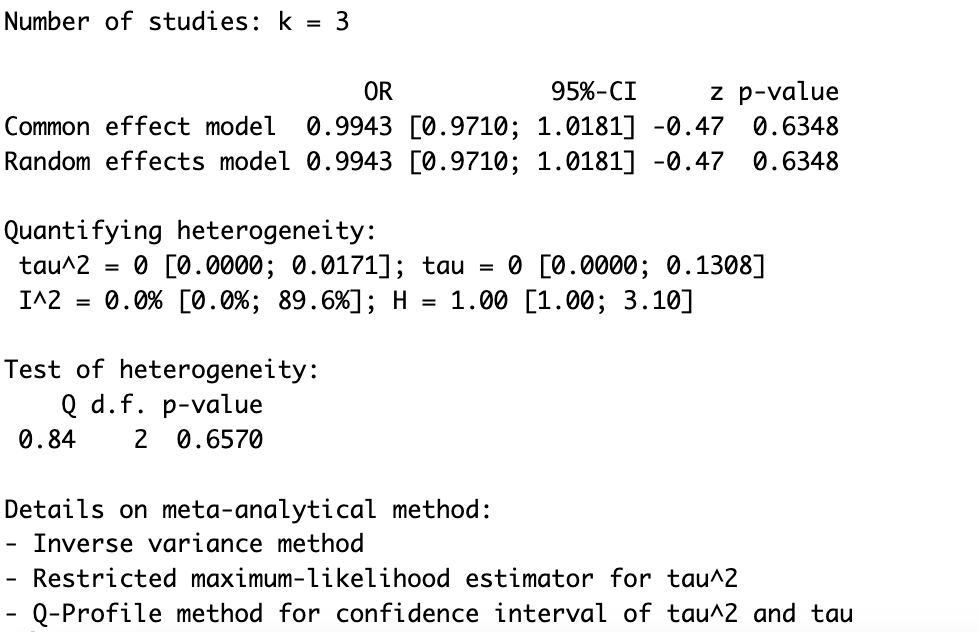


Exposure：Selenium；Outcome：FinnGen(childhood asthma)-30929738(childhood asthma)-34594039(childhood asthma)


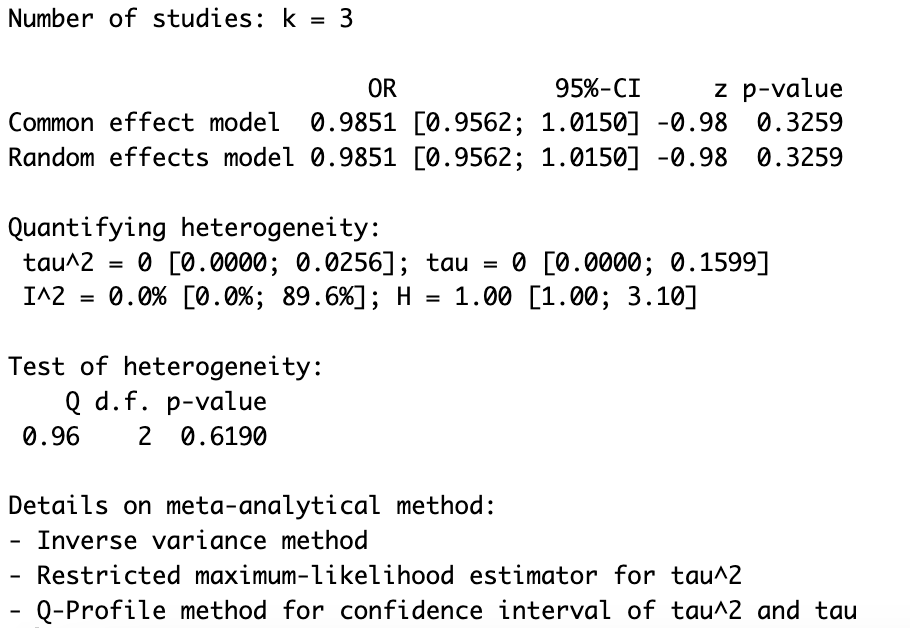


Exposure：Zinc；Outcome：FinnGen(childhood asthma)-30929738(childhood asthma)-34594039(childhood asthma)


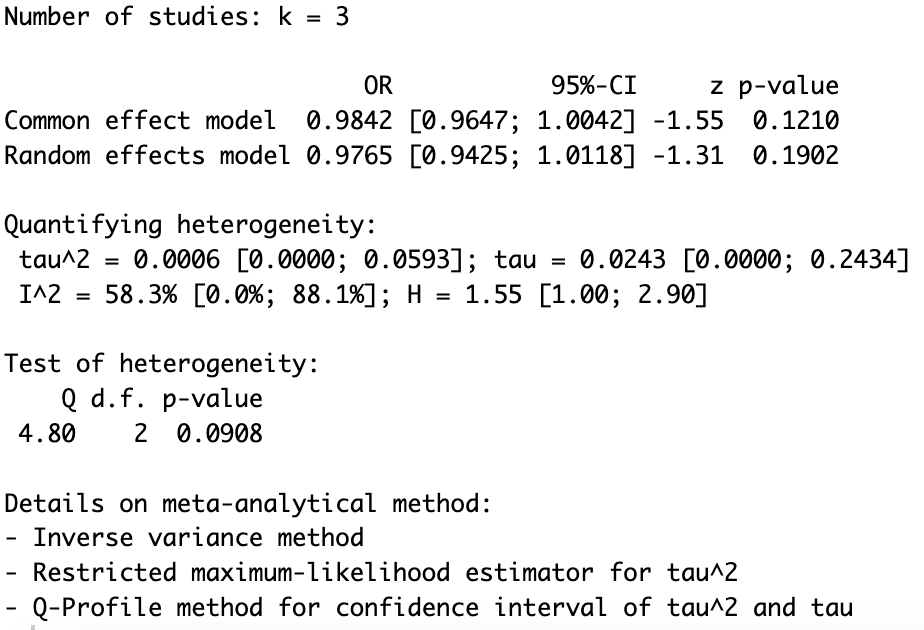


Exposure：Vitamin A (retinol)；Outcome：FinnGen(childhood asthma)-30929738(childhood asthma)-34594039(childhood asthma)


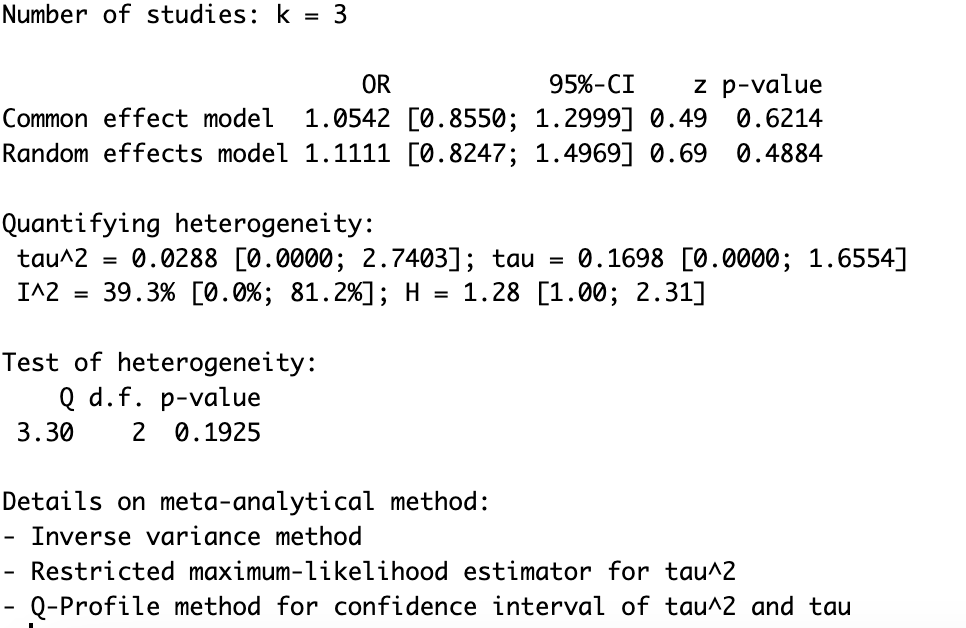


Exposure：Folate；Outcome：FinnGen(childhood asthma)-30929738(childhood asthma)-34594039(childhood asthma)


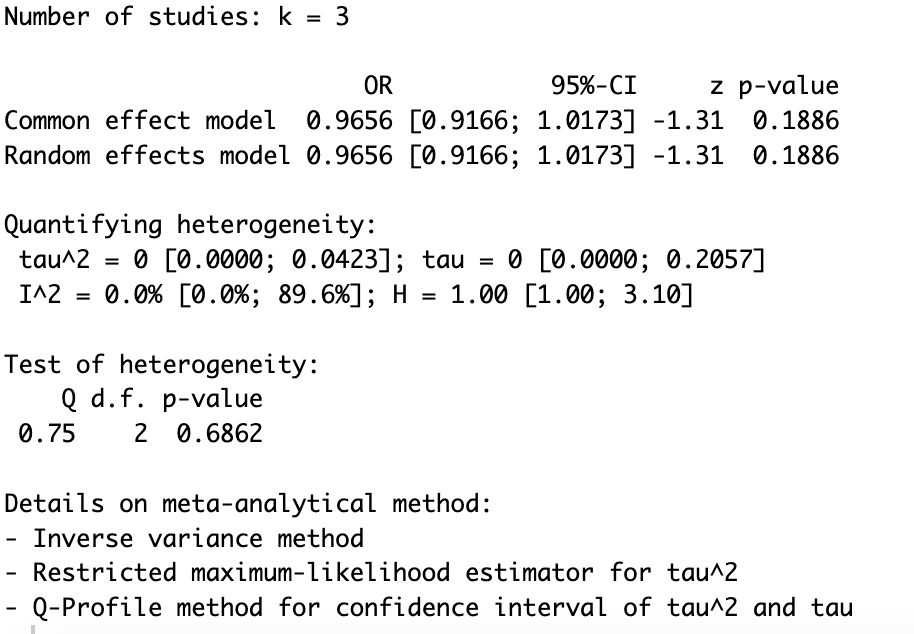


Exposure：Vitamin B12；Outcome：FinnGen(childhood asthma)-30929738(childhood asthma)-34594039(childhood asthma)


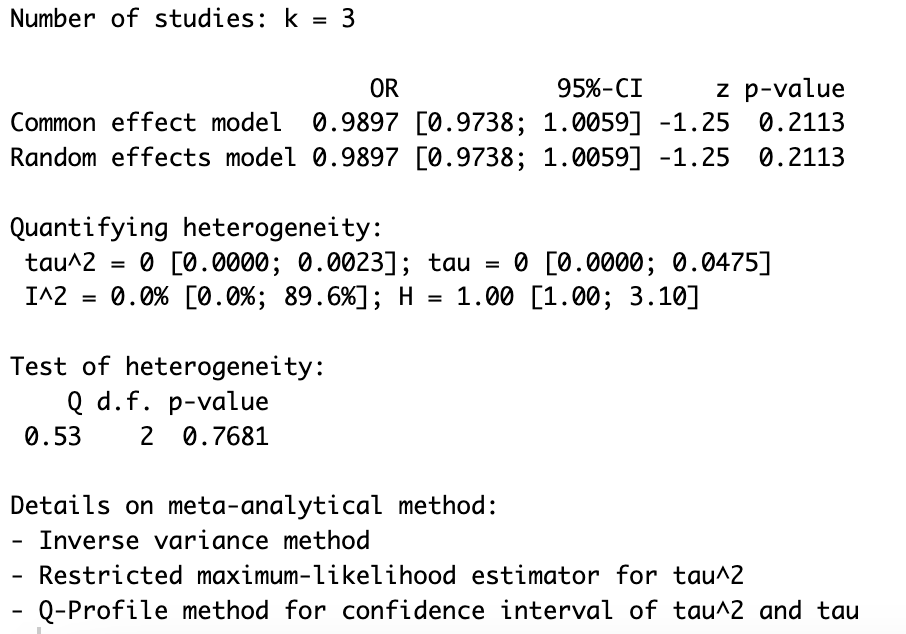


Exposure：Vitamin B6；Outcome：FinnGen(childhood asthma)-30929738(childhood asthma)-34594039(childhood asthma)


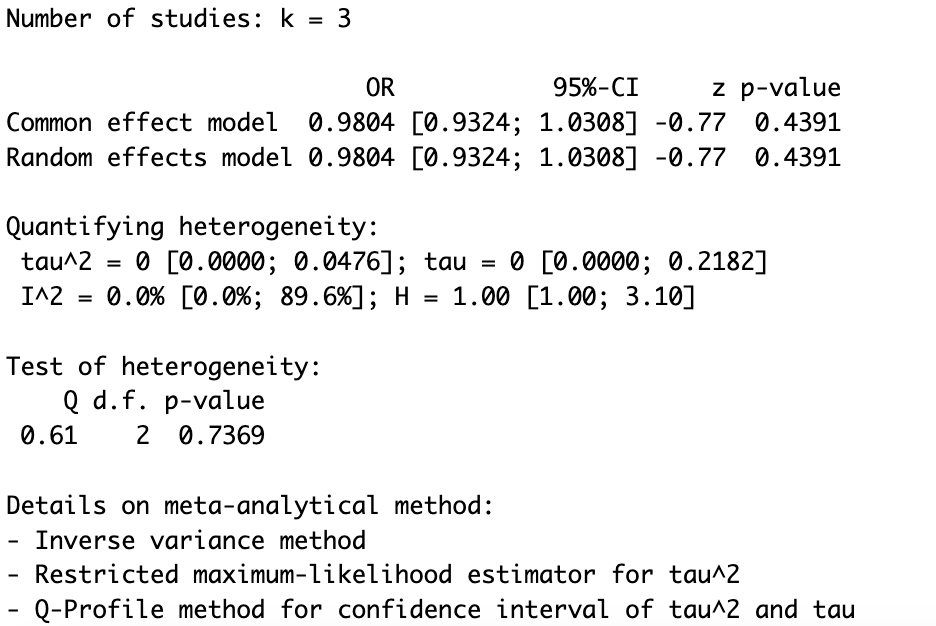


Exposure：Vitamin C；Outcome：FinnGen(childhood asthma)-30929738(childhood asthma)-34594039(childhood asthma)


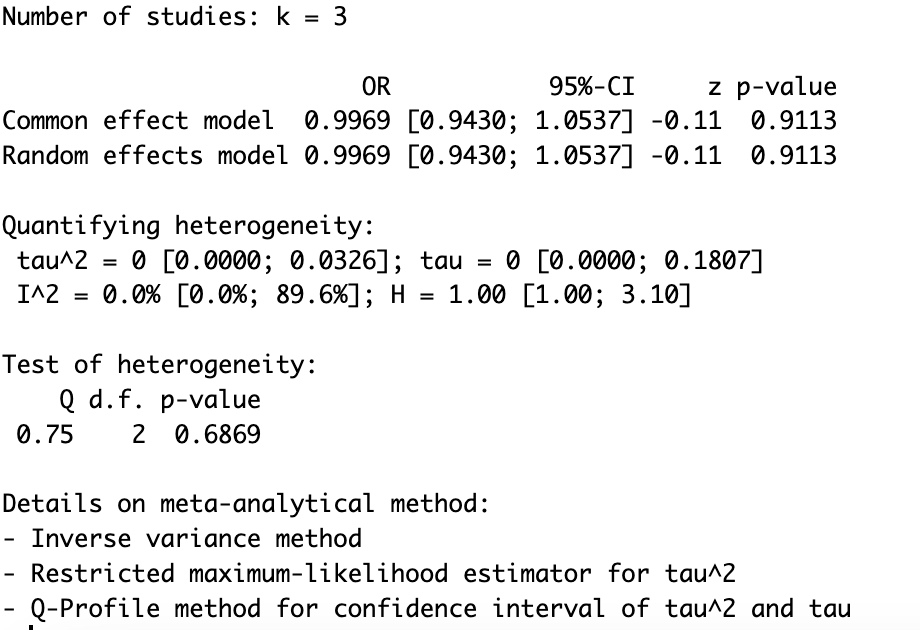


Exposure：Vitamin D；Outcome：FinnGen(childhood asthma)-30929738(childhood asthma)-34594039(childhood asthma)


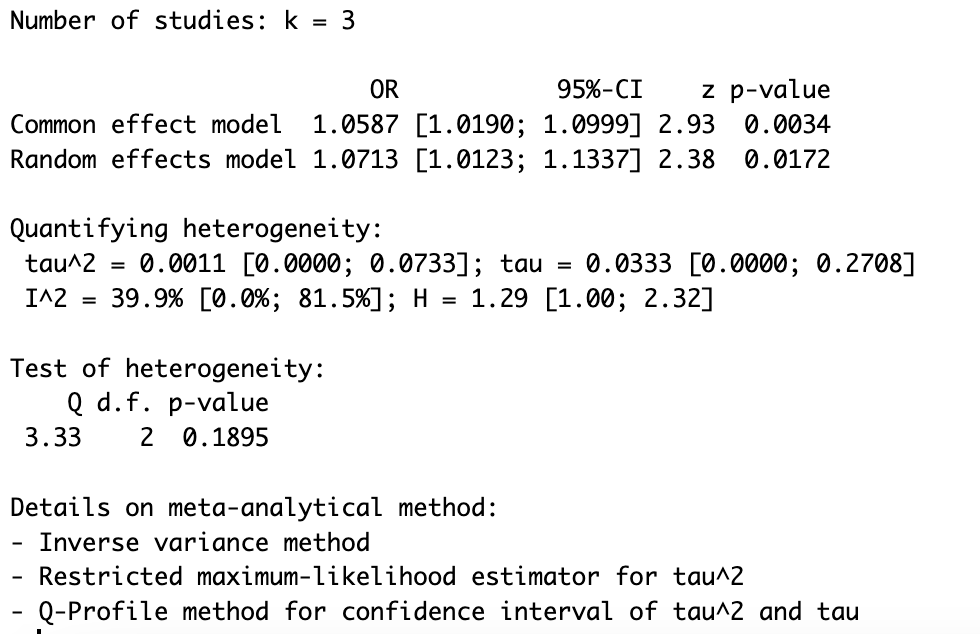


Exposure：Vitamin E；Outcome：FinnGen(childhood asthma)-30929738(childhood asthma)-34594039(childhood asthma)


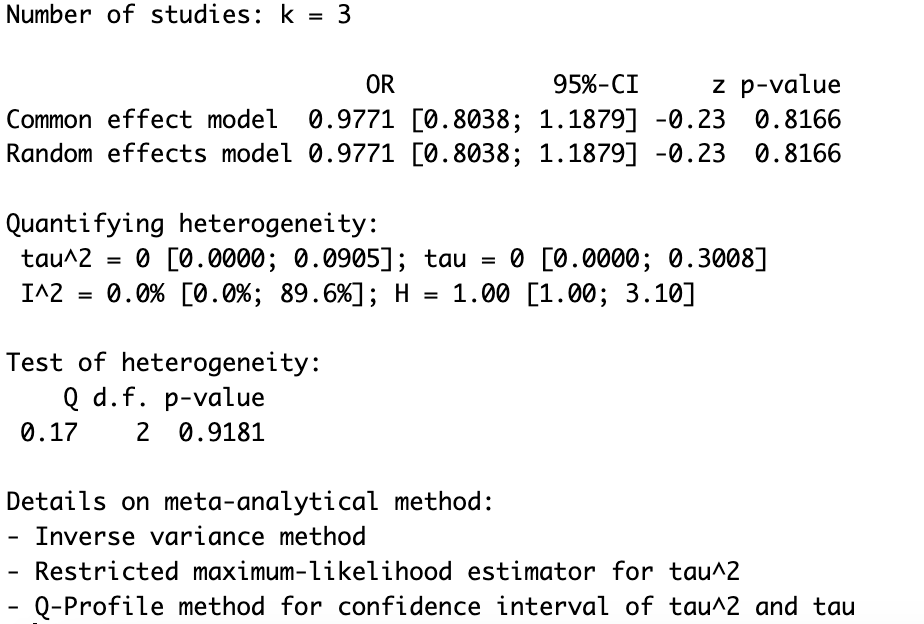

Supplement: Supplementary file 2 [file Table_2.DOCX]
